# Eosinophilic Esophagitis Occurring After Switching to Ultra-Pasteurized Milk: Coincidence or Unrecognized Etiologic Trigger?

**DOI:** 10.7759/cureus.9828

**Published:** 2020-08-18

**Authors:** Augustine Manadan, Ehizogie Edigin, Bashar Attar

**Affiliations:** 1 Rheumatology, John H. Stroger, Jr. Hospital of Cook County, Chicago, USA; 2 Internal Medicine, John H. Stroger, Jr. Hospital of Cook County, Chicago, USA; 3 Gastroenterology and Hepatology, Rush University Medical Center, Chicago, USA; 4 Gastroenterology and Hepatology, John H. Stroger, Jr. Hospital of Cook County, Chicago, USA

**Keywords:** eosinophilic esophagitis, milk, ultra-pasteurized

## Abstract

Eosinophilic esophagitis (EoE) is an increasingly common cause of dysphagia, food impaction, and abdominal pain. Cow’s milk is a major trigger of EoE, but the exact mechanism remains unclear. We present a case of EoE occurring shortly after switching from regularly pasteurized milk to ultra-pasteurized milk.

## Introduction

Eosinophilic esophagitis (EoE) is a chronic immune-mediated disease of the esophagus [1,2]. It is an increasing cause of dysphagia, food impaction, and abdominal pain in both children and adults. EoE is triggered by the ingestion of certain foods primarily cow's milk, wheat, eggs, soy, nuts, and/or seafood [1,2]. EoE is defined as >15 eosinophils per high power field (Hpf) on esophageal epithelial biopsy [1]. Endoscopic features of EoE include linear furrows, circular ridges, rings, white microabscesses, and strictures [1]. Cow’s milk is one of the major triggers of EoE [3], but the exact mechanism remains unclear. We describe a case of EoE occurring shortly after switching from regularly pasteurized milk to ultra-pasteurized (UP) milk.

## Case presentation

**Figure 1:**
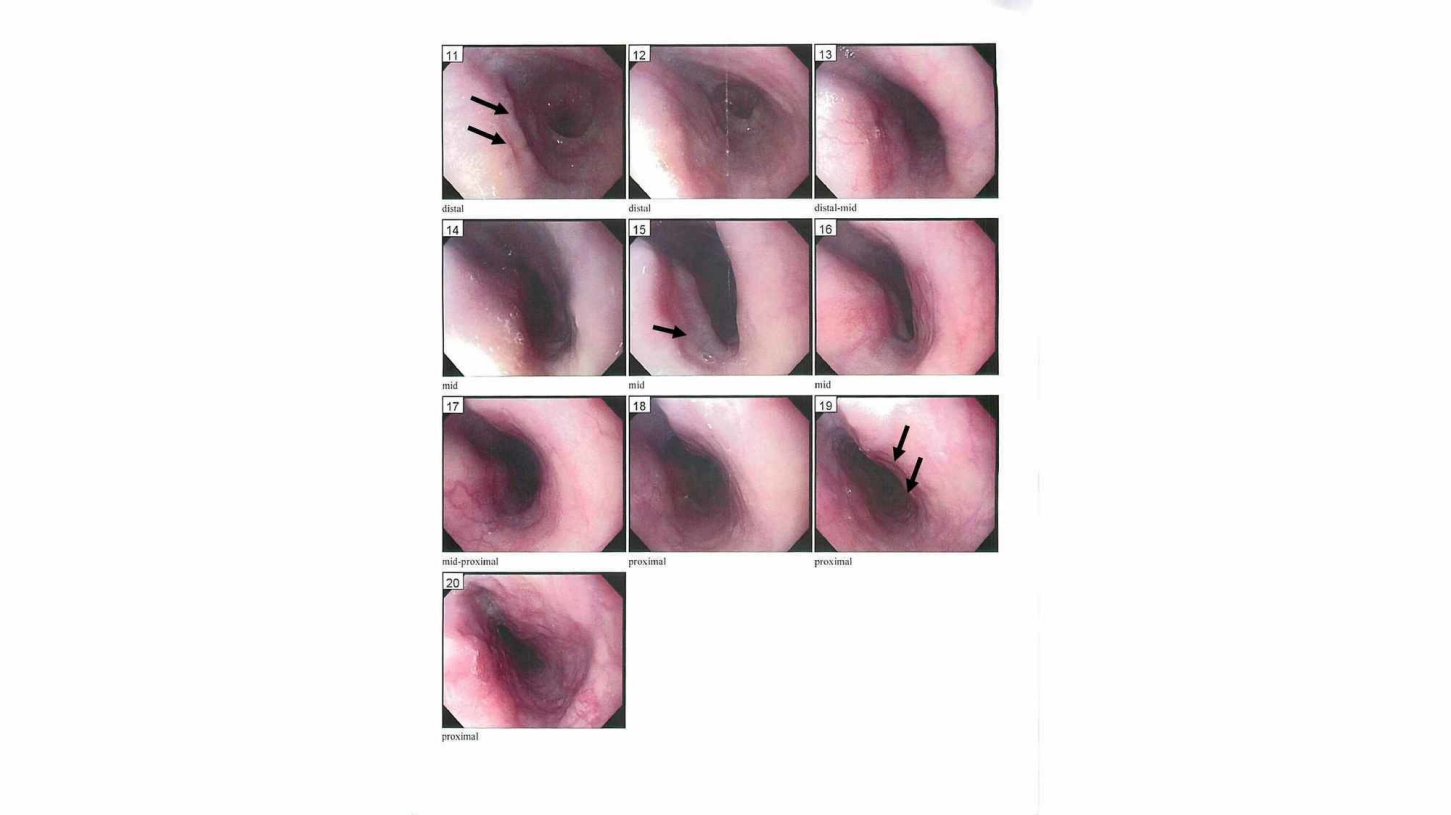
Esophagogastroduodenoscopy showing mild exudate and furrows throughout the esophagus, most notable distally 11: distal esophagus with furrows, 15: mid-esophagus with edema and exudate, 19: proximal esophagus with mild furrows

Our case was a nine-year-old boy with a past medical history significant for atopic dermatitis, seasonal allergic rhinitis, tree nut allergies, and mild reactive airway disease who developed episodes of abdominal pain, intermittent nausea, occasional vomiting, and post-prandial cough. He visited the emergency room twice for abdominal pain without the identification of the source of the pain. His physical examination was completely normal. He was started on twice-daily proton pump inhibitor, and esophagogastroduodenoscopy (EGD) three months later showed mild exudate and furrows throughout the esophagus, which were most notable distally (Figure 1).

**Figure 2:**
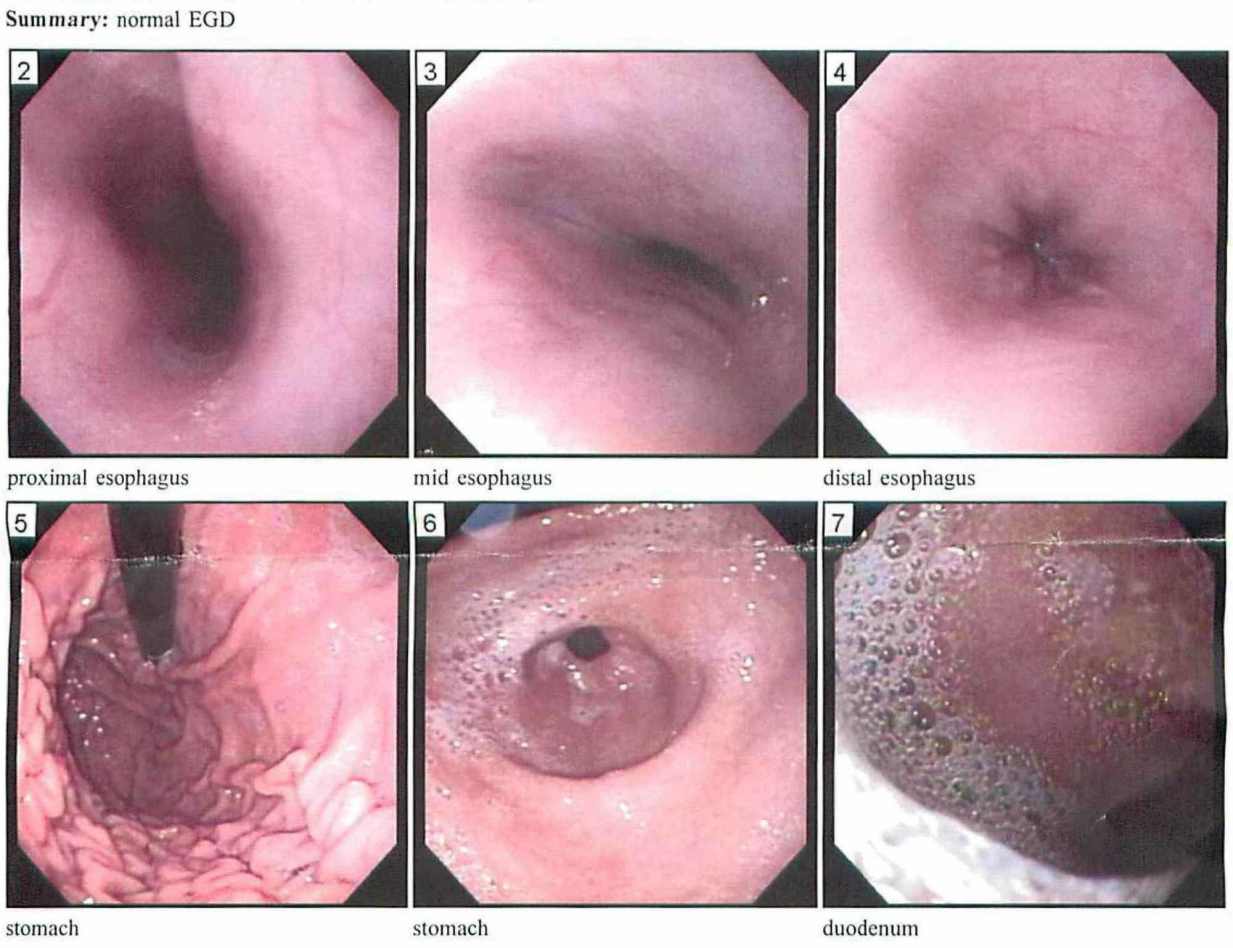
Repeat esophagogastroduodenoscopy showing a grossly normal esophagus Normal esophagogastroduodenoscopy with an unremarkable esophagus, duodenum, and stomach

The microscopic exam revealed >100 eosinophils per Hpf in both middle and distal esophagus and 60-65 eosinophils per Hpf in the proximal esophagus. He was diagnosed with EoE, and gastrointestinal (GI) symptoms resolved shortly after starting swallowed fluticasone 220 mcg two puffs twice daily. Repeat EGD four months later showed grossly normal esophagus, and repeat esophageal biopsy showed no eosinophils (Figure 2).

Interestingly, his first GI symptom was a prolonged episode of nausea, which developed one week after his household switched from regularly pasteurized cow’s milk to UP organic 2% cow’s milk. No medical evaluation was conducted at that time point, but, in retrospect, it is believed that this initial nausea was due to evolving EoE. The parents carefully recorded his dietary history after this first episode of nausea because of a remote history of tree nut anaphylaxis. No soy, nuts, or seafood were consumed during this period. His only previous exposure to UP milk occurred one to two times per year in the form of children’s non-refrigerate single-serve milk boxes. In the two months following the household switch to UP milk, he progressively developed worsening abdominal pain, nausea vomiting, and postprandial cough. Symptoms improved with the commencement of treatment for EoE and switching back from UP to regularly pasteurized milk consumption. Later, when he has been off swallowed corticosteroids, even regularly pasteurized milk repeatedly exacerbated his GI symptoms.

## Discussion

UP milk has been growing in the marketplace over the last several decades. UP milk’s major advantage is its extended shelf-life (around 30 days) under refrigeration conditions compared with regularly pasteurized milk, which typically has a shelf-life of around 10 days [4]. In the USA, ultra-pasteurization is defined as the process of heating raw milk to ≥138 °C for ≥2 seconds. In regular pasteurization, milk is only heated to ≥72 °C for ≥15 seconds [4]. UP milk differs from ultra-high temperature (UHT) treated milk, which is typically processed at around 140 °C for around 4 seconds. UHT-treated milk can be stored at room temperature and has a shelf-life of more than six months. The heat treatments used in ultra-pasteurization and UHT treatment are known to alter milk's biochemical properties [4,5]. These biochemical changes are believed to explain the altered taste, smell, color, nutrient levels, and microbiology of the extended shelf-life milk [4-7]. Following is a basic summary of the described biochemical changes resulting from ultra-pasteurization. First, β-lactoglobulin (whey protein) is denatured at a much greater extent in ultra-pasteurization than in typical pasteurization, and some cases reach up to 61% of the protein [4]. UP causes β-lactoglobulin to unfold (denature) such that the buried sulfhydryl groups, normally masked in the native protein, are exposed to the outer surface. Second, ultra-pasteurization initiates the Maillard reaction in which lactose reacts with the milk proteins. The extent of this is often measured as furosine. Furosine levels are often used as a measure of heat load in milk [4]. Third, lactulose is formed from lactose during heating. Lactulose levels are a good measure of heat treatment, and its levels are increased with ultra-pasteurization [4]. Fourth, ultra-pasteurization increases the thiol content when compared to conventionally pasteurized milk [4]. Volatile sulfur compounds containing the thiol group are largely responsible for the cooked, sulfur flavor in high-temperature treated milk [7].

In the case of UHT milk, major reductions in microRNA (miRNA) levels are described [8]. Kirchner et al. propose that miRNA levels, specifically in the fat fraction, may explain the beneficial effect of raw farm milk consumption on asthma and allergic disease [8]. Additionally, Loss et al. showed that in contrast to UHT milk, raw milk consumption during the early years of life was inversely associated with the occurrence of upper respiratory tract infections [9]. These data support the concept that heat-sensitive components such as proteins present in raw milk may have a beneficial effect on the immune system.

Despite the biochemical changes produced from heating milk above regular pasteurization temperatures, there is no published scientific evidence known to the authors to date that these changes increase milk’s allergenicity. In fact, in the case of immunoglobulin E (IgE)-mediated milk allergy, some studies show a reduction in allergenicity of UHT proteins [10]. Additionally, boiled and baked milk processed at temperatures well above UHT range has been successfully given to infants with IgE milk protein allergy [11]. Thus, it would be biologically plausible to conclude that UP or UHT actually serves a beneficial role in allergic disease, but we believe that it is not accurate to extrapolate results of IgE-mediated milk allergy studies and apply them to a Th2 disease such as EoE [12,13]. The traditional IgE-mediated skin prick test has not been effective in identifying the causative food in EoE [14].

We hypothesize that partially or abnormally denatured UP milk proteins could be seen as immunologically foreign upon entering the bloodstream in a genetically prone host; once sensitized to milk proteins, it could be difficult to tolerate any type of milk, as seen in our patient.

A possible limitation to our hypothesis is that the boy had an allergic history before switching to UP milk, which would favor a coincidence rather than causation. Additionally, heat treatment of proteins would intuitively seem to approach an elemental diet, which is one of the most effective treatments of EoE.

It is difficult to get an exact history of UP milk products in the American marketplace, but both UP and UHT milk products were introduced over the last several decades to extend shelf life. In the case of UHT, it has been described in the scientific literature since the 1960s and has been common in the European marketplace for many years. Michael Janofsky wrote in a 1993 New York Times article about the first large introduction of UHT milk to the USA market by an Italian company called Parmalat after they bought an American dairy producer and began producing UHT milk in a plant in Grand Rapids, Michigan [15]. Despite the absence of exact marketing data, it appears that both UP and UHT dairy product consumption has greatly increased over the last 25 years in the form of milk, whipping creams, and coffee creamers. Interestingly, mass commercial adoption of UP and UHT dairy products in the USA roughly parallels the time frame in which EoE has emerged as a medical problem. Additionally, multiple studies using food elimination diets as a treatment of EoE and followed by sequential food reintroduction has implicated dairy as a major culprit [2].

## Conclusions

In summary, UP and UHT are processes used to prolonged shelf life, but the high heats biochemically alter milk. Cow’s milk has been consumed for thousands of years, but EoE is a relatively new illness and roughly parallels the introduction of UP and UHT milk products. We question if the newer methods of processing milk are at the genesis of EoE by altering protein structure and their allergenicity. This report of EoE occurring temporally after the introduction of UP milk is not conclusive evidence of causality, but it may warrant further observational study.
